# Genome-wide analysis of the *Solanum tuberosum* (potato) trehalose-6-phosphate synthase (TPS) gene family: evolution and differential expression during development and stress

**DOI:** 10.1186/s12864-017-4298-x

**Published:** 2017-12-01

**Authors:** Yingchun Xu, Yanjie Wang, Neil Mattson, Liu Yang, Qijiang Jin

**Affiliations:** 1 College of Horticulture, Nanjing Agricultural University, Nanjing, 210095 China; 2 Horticulture Section, School of Integrative Plant Science, Cornell University, 134A Plant Science Bldg, Ithaca, NY 14853 USA; 3 Institute of Plant Protection, Jiangsu Academy of Agricultural Sciences, Nanjing, 210095 China

**Keywords:** *Solanum tuberosum*, Trehalose-6-phosphate synthase, gene family, expression profiling

## Abstract

**Background:**

Trehalose-6-phosphate synthase (TPS) serves important functions in plant desiccation tolerance and response to environmental stimuli. At present, a comprehensive analysis, i.e. functional classification, molecular evolution, and expression patterns of this gene family are still lacking in *Solanum tuberosum* (potato).

**Results:**

In this study, a comprehensive analysis of the *TPS* gene family was conducted in potato. A total of eight putative potato *TPS* genes (*StTPSs*) were identified by searching the latest potato genome sequence. The amino acid identity among eight StTPSs varied from 59.91 to 89.54%. Analysis of d_N_/d_S_ ratios suggested that regions in the TPP (trehalose-6-phosphate phosphatase) domains evolved faster than the TPS domains. Although the sequence of the eight *StTPSs* showed high similarity (2571-2796 bp), their gene length is highly differentiated (3189-8406 bp). Many of the regulatory elements possibly related to phytohormones, abiotic stress and development were identified in different *TPS* genes. Based on the phylogenetic tree constructed using *TPS* genes of potato, and four other *Solanaceae* plants, *TPS* genes could be categorized into 6 distinct groups. Analysis revealed that purifying selection most likely played a major role during the evolution of this family. Amino acid changes detected in specific branches of the phylogenetic tree suggests relaxed constraints might have contributed to functional divergence among groups. Moreover, *StTPSs* were found to exhibit tissue and treatment specific expression patterns upon analysis of transcriptome data, and performing qRT-PCR.

**Conclusions:**

This study provides a reference for genome-wide identification of the potato *TPS* gene family and sets a framework for further functional studies of this important gene family in development and stress response.

**Electronic supplementary material:**

The online version of this article (10.1186/s12864-017-4298-x) contains supplementary material, which is available to authorized users.

## Background

Trehalose is a non-reducing disaccharide and known as a quantitatively important compatible solute in distinct organisms, for example, bacteria, fungi, algae, and plants [1–3]. Recent accumulating evidence has caused great interest in trehalose, due to its role as a potential signal metabolite and a cell stabilizer in plants. Trehalose is believed to interact with pathogens and herbivorous insects in plants as well as protect plants from various environmental stresses, i.e. heat, cold, desiccation, freezing, hypoxia and oxidative stress [4, 5]. A striking example is in “resurrection plants”, e.g. *Selaginella lepidophylla*, *Myrothamnus flabellifolius* and *Sporobolus spp*., which survive under extreme desiccation, where up to 99% of their water has been removed. The protective effects of trehalose can be explained by water replacement hypothesis or the glass transition hypothesis [4]. Under water deficiency, resurrection plants accumulate massive amounts of trehalose reaching levels up to 10 –20% of the dry weight [6] which enable them to persist in metabolic stasis for several years until re-watered. However, it is interesting to note that trehalose levels are much lower in crops plants.

It is well established that an important enzyme, trehalose-6-P synthase (TPS), catalyzes the conversion of Glc-6-P and UDP-Glc into trehalose-6-P (T-6-P) [7]. T-6-P is then catalyzed by T-6-P phosphatase (TPP) and releases trehalose. Both plants and yeast (*Saccharomyces cerevisiae*) share a similar biosynthesis pathway [7, 8]. So far, TPS proteins have been purified from several organisms, including *S. lepidophylla* [9, 10], yeast [11], *Mycobacterium smegmatis* [12], and *Mycobacterium tuberculosis* [13]. Among these organisms, the biosynthesis of trehalose in *Escherichia coli* and *Saccharomyces cerevisiae* has been well studied. It was found that the TPS are specific for either UDP-glucose or GDP-glucose as the glucosyl donor [8, 14]. Further studies indicated that T-6-P could restrict glucose influx by its interaction with sugar kinase activities and glucose transport [15].

In spite of low trehalose content in plants, recent evidence showed that expression or overexpression of *TPS* genes in some plants, i.e. tobacco, could lead to pounced changes on growth performance and morphology under drought stress [16, 17]. In *Selaginella*, studies suggest involvement of a functional TPS (SlTPS1) in regulating plant response to heat and salt stresses [18, 19]. In fact, it has become clear that overexpression or expression of *TPS* genes conferred biotic and abiotic stress tolerance of transgenic plants [7, 17, 20, 21]. Despite this, there is no evidence that the enhanced tolerance in these plants is associated with changes of trehalose content [21]. In wheat and cotton, water deficiency only triggers a slight increase in trehalose content [41]. Whether these observed effects on stress tolerance in these transgenic lines were attributed to small changes in trehalose levels [7] has so far been poorly described.

A large number of putative trehalose synthesis genes have been identified and characterized in a wide range of plants [22–24]. In *Arabidopsis*, studies identified 11 *TPS* gene family members, defined by the presence of conserved TPS and TPP domains and can be categorized into two main subfamilies [25]. It is now well accepted that the plant *TPS* gene family is a large gene family with multiple copies, and known to participate in a great array of biological processes [24, 26, 27]. Other functions have been attributed to *TPS* genes. For example, TPS has been found to act as a sucrose signal for trehalose in stress response. Notably, the *Arabidopsis TPS* gene, *AtTPS2*, was demonstrated to be a regulator of glucose, abscisic acid and stress signaling [28]. Further, T6P is also recognized as a regulator of sugar metabolism in plants [29–31]. T6P was found to inhibit the effect of SNF1-related protein kinase1, which is a central integrator of stress and metabolic signals, to regulate plant growing tissues [30]. However, their actual functions in higher plants are largely unknown, particularly those involved in important signaling pathways. Identification and characterization *TPS* gene family members is particularly relevant for understanding the role of TPS in plants, both for genetic diversity to obtain a broader understanding of the function of TPS, and as a potential gene resource for improving crop plant defense against biotic and abiotic stresses.

Potato is an important food and economic crop globally. *TPS* genes in potato have not been well characterized previously. This study investigated the distribution of *TPS* genes from whole genome-wide resources, genetic structure of *TPS* genes in potato genomes, and expression patterns of the gene family members in different tissues or under various stresses. The evolutionary characterization of the *TPS* gene family in potato, and four other *Solanaceae* plants including tomato, pepper, tobacco and petunia were also examined. These results contribute to a better understanding of potato *TPS* gene family, and facilitate further functional studies of them.

## Results and discussion

### Identification of the potato *TPS* gene family members

**Fig. 1:**
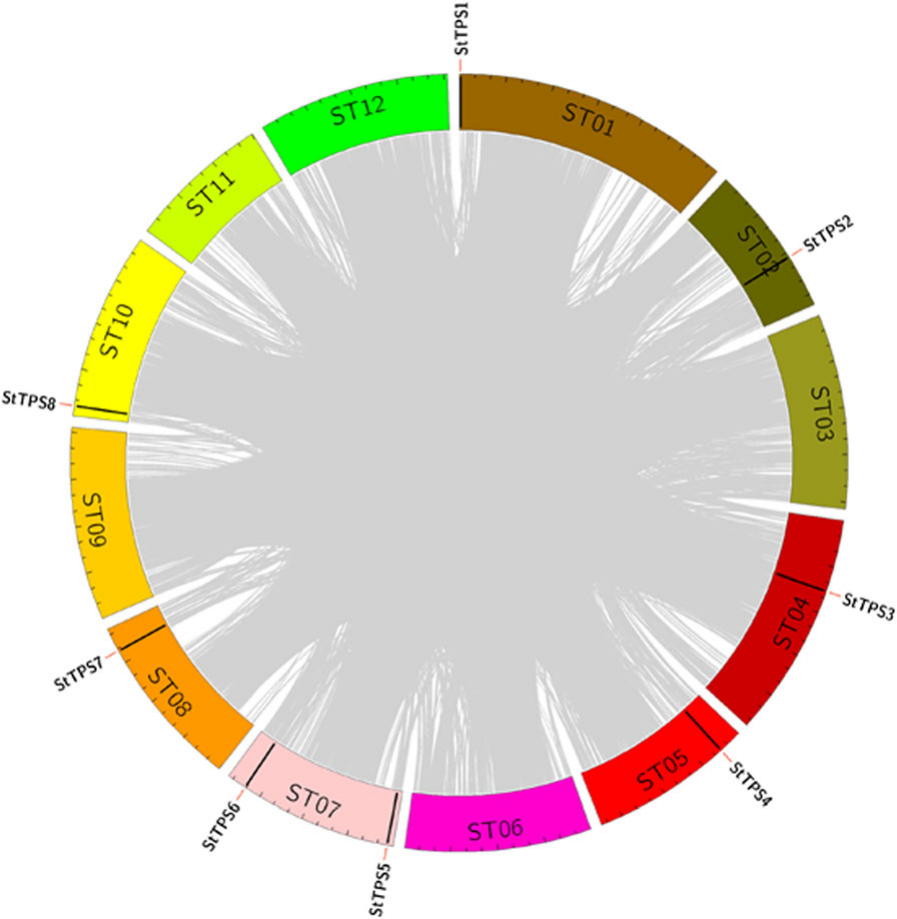
Chromosomal locations and synteny analysis of *StTPS* genes. Gray background lines indicate collinear blocks in respective genome

The potato is an important dicotyledonous source of human food. Compared with other important crops, i.e. rice, there is relatively less genetic research on potato. Trehalose protects bioactive substances and cell structures of cells against various environmental stresses [2, 3, 18, 20, 21]. Trehalose-6-phosphate synthases (TPSs), important enzymes in the biosynthesis of trehalose, have emerged recently as key players in protecting plants from heat, nutrient, osmotic, and dehydration stress, as well as toxic chemicals. Of particular interest, TPSs might function as regulatory molecules in linking trehalose metabolism to glucose transport and glycolysis [3]. However, very little research has focused on the identity and function of potato *TPS* genes. In this work, the latest version of the potato genome was downloaded to identify genes encoding TPS using HMMER (v3.1) [32] with HMMs of TPS and TPP domains. While the initial screen identified 11 ORFs predicted to encode putative TPS proteins, only 8 contain both TPS and TPP domain and were identified as TPS protein (Table 1). Previous studies have identified 11 *TPS* genes in *Arabidopsis*, 11 in rice, 12 in *Populus trichocarpa* and 13 in soybean [33]. Our data suggest a loss in *TPS* genes in potato as compared to the *TPS* gene family in *Arabidopsis*, rice, soybean, and *Populus trichocarpa*. To determine the genomic distribution of *StTPS* genes, we noted their position on each chromosome based on the information obtained from the genome database (Fig. 1). It was found that *StTPS* genes were dispersed in seven potato chromosomes. Eight potato *TPS* genes were designated as *StTPS1*–*StTPS8* according to their order on the chromosomes. Synteny blocks were analyzed among the potato chromosomes for further investigation of the possible evolutionary mechanism of *StTPS* genes in potato (Fig. 1). It is interesting to note that none of the *StTPS* gene pairs were observed within a synteny block, which indicating that *StTPS* genes were duplicated by other modes but not segmental, tandem and proximal, and *StTPS* gene family might expand after two previously reported whole-genome duplication events [34]. The eight predicted full length TPS proteins varied from 857 to 932 amino acid residues and the relative molecular mass ranged from 94.877 to 105.815 kDa, with isoelectric points in the range of 5.520 to 6.390. Subcellular localization prediction suggested that most of the StTPSs might be located in cytoplasm, while only a few might be located in plasma membrane or nucleus (Table 1). Subcellular localization of a gene product is closely related to its functional involvement.

**Table 1:** List of the *StTPS* genes identified in this study

Gene ID	Gene Accession Number	CDS (bp)	Deduced Polypeptide				Predicted Subcellular localization
			Length (aa)	MW (kDa)	pI	GRAVY	
StTPS1	PGSC0003DMG400022778	2574	857	97.354	5.610	-0.218	Cytoplasmic
StTPS2	PGSC0003DMG400028467	2553	850	96.178	5.700	-0.229	Cytoplasmic
StTPS3	PGSC0003DMG400023995	2574	857	96.528	5.520	-0.188	Cytoplasmic
StTPS4	PGSC0003DMG400004114	2760	919	103.894	5.940	-0.131	Nuclear
StTPS5	PGSC0003DMG400027449	2625	874	98.541	6.390	-0.239	Cytoplasmic
StTPS6	PGSC0003DMG400017276	2517	838	94.877	5.630	-0.280	Cytoplasmic
StTPS7	PGSC0003DMG401017546	2589	862	97.255	5.900	-0.172	Cytoplasmic
StTPS8	PGSC0003DMG400010556	2799	932	105.815	5.500	-0.211	Plasma Membrane

### Multiple sequence alignment

**Fig. 2:**
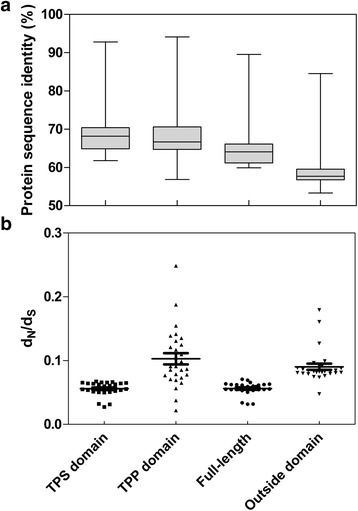
Pairwise sequence identities and d_N_/d_S_ ratios for different regions of potato TPS proteins. Pairwise sequence identities (**a**) and dN/dS ratios (**b**) between TPS domain, TPP domain, full length protein sequence and sequence outside domain were calculated

To clarify the characteristics of *TPS* gene family in potato, multiple sequence alignment of amino acid sequences was performed using Clustalx (Additional file 1: Figure S1). The results showed that the catalytic centers in StTPSs are highly conserved, implying the corresponding genes encode active TPS enzymes. The amino acid identity among eight StTPSs ranged from 59.91 to 89.54%, with the highest identity between StTPS3 and StTPS4 (Fig. 2a), and the lowest identity between StTPS5 and StTPS8. Relatively high divergence was observed in some regions of the amino acid sequences outside of the TPS and TPP domain. The average identity of amino acid sequence of TPS and TPP domains were about 70%, while it was only 60% of the sequences outside domains. It seems likely that these non-conserved regions may contribute largely to functional distinction.

We then analyzed substitution rate ratios of the synonymous substitution rate (d_S_) versus the non-synonymous substitution rate (d_N_) of *StTPSs*, as this could measure selection pressure on amino acid substitutions, and reflecting whether Darwinian positive selection was involved in driving gene divergence after duplication. Results in Fig. 2b showed that all the estimated d_N_/d_S_ values of different domains and regions outside domains were substantially less than 1. Generally, d_N_/d_S_ ratio >1 indicates positive selection, a ratio <1 indicates negative or purifying selection and a ratio = 1 indicates neutral evolution [35, 36]. This suggested that potato *TPS* gene family might have undergone purifying selection.

In contrast to the result of protein sequence identity, the d_N_/d_S_ ratios in TPP domains were much higher than in TPS domains as well as regions outside TPS domains (Fig. 2b). This observation revealed that the sequence of TPP domains evolved faster than the TPS domain, which might be caused by relaxed purifying or positive selection in the TPP domain. The positive selective effect on residues of TPP domains might ultimately lead to changes in protein function.

### Gene structures and protein domains of StTPSs

**Fig. 3:**
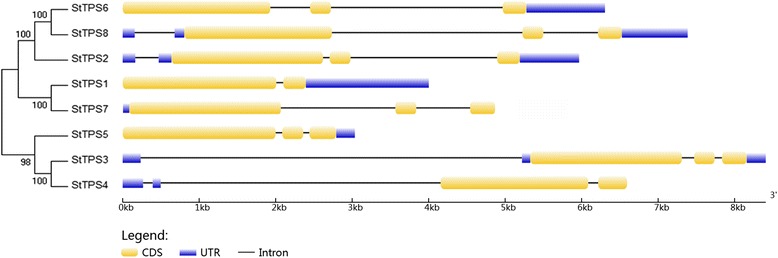
Exon-intron structures of the identified *StTPS* genes. The graphic representation of the optimized gene model displayed using GSDS. Genes were grouped by an unrooted phylogenetic tree resulting from the full-length amino acid alignment of all the StTPS proteins as shown on the left side of the figure

We then analyzed the exon/intron boundaries of *StTPS* genes, as this can provide additional evidence for the evolution of multiple gene families [37]. We observed that except *StTPS1* and *StTPS4*, most genes harbored two introns in the CDS region (Fig. 3). *StTPS* genes identified on the terminal node of the phylogenetic tree were more variable as compared with previous observations on *TPS* gene structure [33]. Moreover, in spite of the high similarity in CDS length (2571-2796 bp) among eight *StTPS* genes, their total gene length is more variable (3189-8406 bp).

**Fig. 4:**
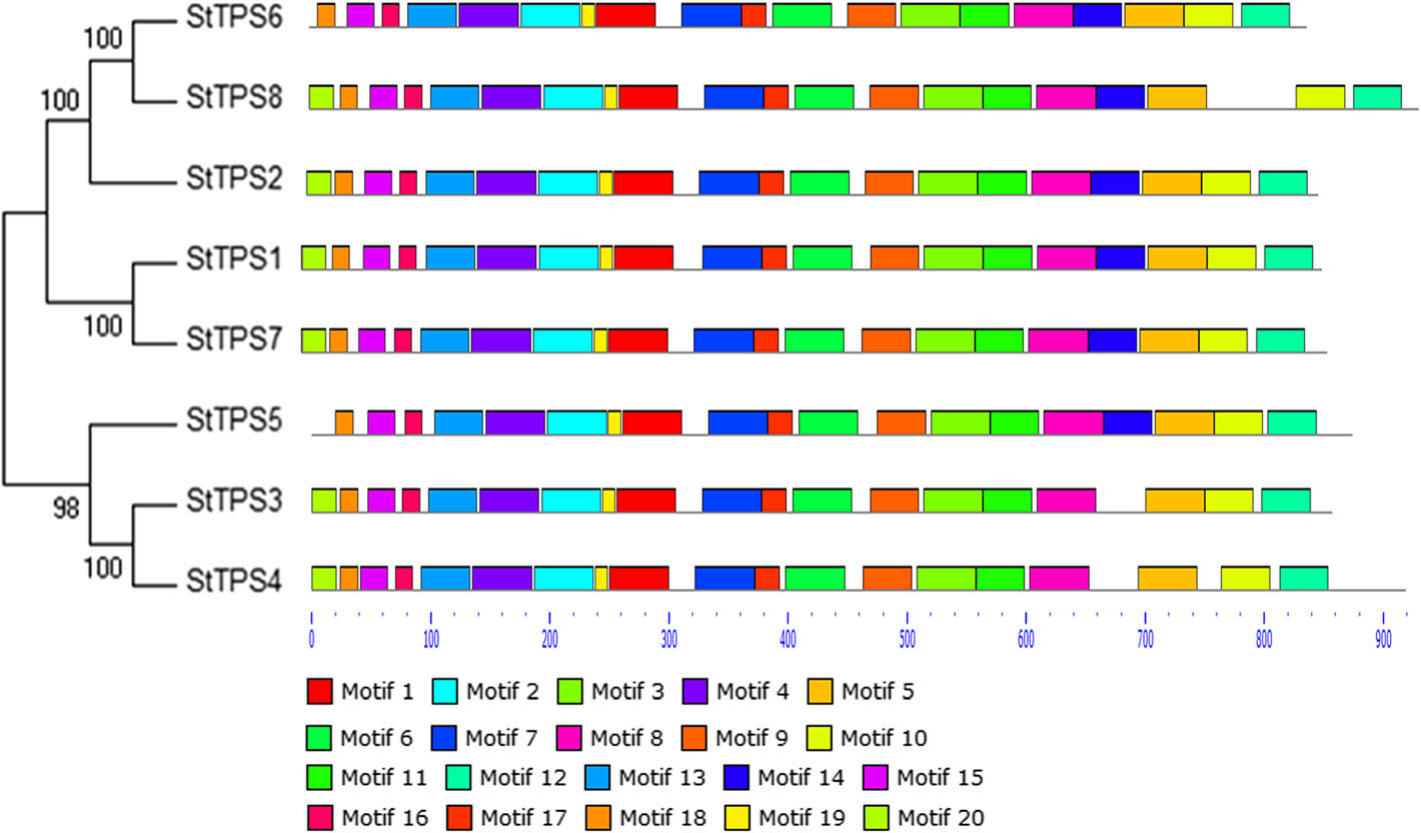
Schematic diagram of amino acid motifs of TPS protein. The different-colored boxes represent different motifs and their position in each TPS sequence

The motif distribution in eight *StTPS* genes was investigated using the MEME program. MEME software identified a total of 20 conserved motifs in *StTPS* as well as their distribution (Fig. 4, Additional file 2: Figure S2). With the exception of three *StTPS* genes, including *StTPS2*, *StTPS3* and *StTPS4*, all motifs were found distributed diffusely among the other five genes. According to Fig. 4, TPS domains are composed of 12 motifs including motif 1, 2, 3, 4, 6, 9, 10, 13, 14, 15, 17 and 20, while TPP domains are composed of 2 motifs including 8 and 19. These features are consistent with those observed in other plants [38].

**Fig. 5:**
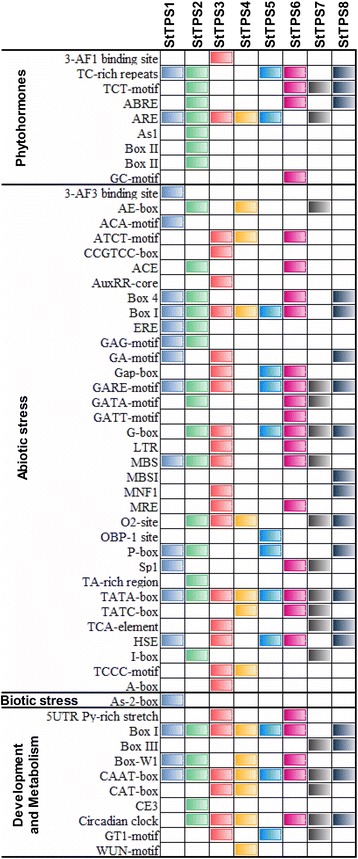
*cis*-regulatory elements in *StTPS* genes. 1.5 kb upstream regions from the translation start codon of the genes were used to predict *cis*-regulatory elements in *StTPS* genes using PlantCare server. The graph was plotted on the basis of presence of *cis*-regulatory element responsive to specific elicitors/conditions

The promoter region regulates expression of genes in response to environmental stimuli. Determining promoter region features, especially the *cis*-acting elements, is key to understanding the systems that regulate gene expression [39]. For instance, ABA-responsive elements (ABREs) regulate gene response to ABA, drought or salt signals [40, 41]. To identify *cis*-regulatory elements in *StTPS* genes, the 1.5 kb upstream region of the eight genes were extracted from the potato genome and analyzed using PlantCare server (Fig. 5). Several regulatory elements predicted in *StTPS* promoters were associated with phytohormones, abiotic stress and developmental processes. Further, we also identified a biotic stress response element (As-2-box) in *StTPS1*. These predicted cis-regulatory elements were evenly distributed throughout the promoter regions of the *StTPS* genes (Fig. 5). The presence of hormone-responsive elements (abscisic acid, auxin, gibberellin, salicylic acid and Jasmonic acid) could be interpreted as an indication that these *TPS* genes might be involved in various signaling pathway of phytohormones. In particular, *StTPS2* contained the largest number of phytohormones-responsive elements, suggesting an important role in phytohormone response. *StTPS* genes were predicted to contain various abiotic stress-responsive elements, most of which were involved in plant response to environmental stimuli. For example, *StTPS3* were found induced during both anaerobic and dark condition, indicating that it might involve in plant submergence response. *StTPS1* and *StTPS2* contain regulatory elements responsive to low temperature. These conclusions were supported by several reports that *TPS* expression levels increased under drought [42], salt and temperature stresses [18, 43] in various plants.

### Evolution analysis of *TPS* genes

**Fig. 6:**
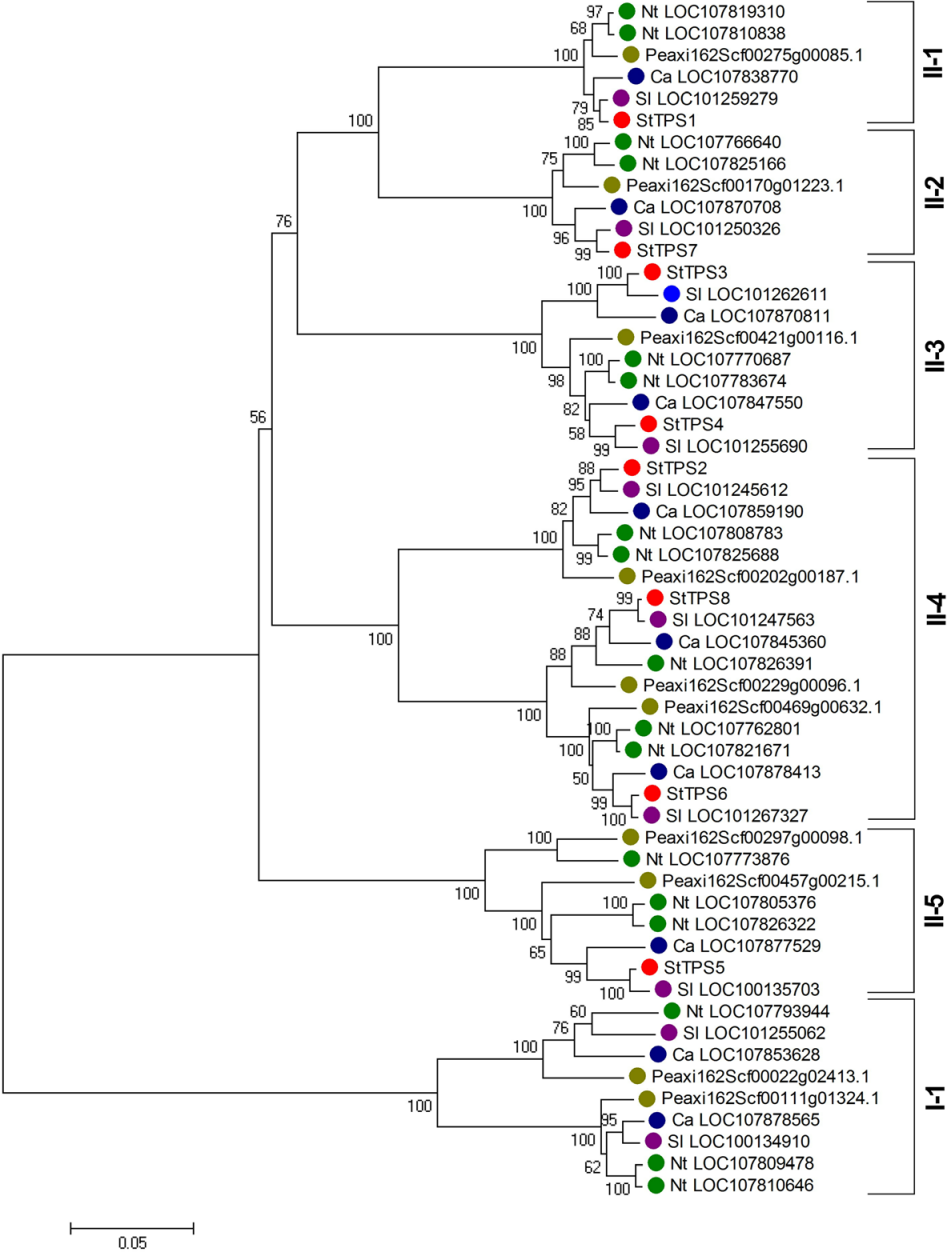
Phylogenetic relationships of the TPS gene family members from potato (StTPS), tomato, pepper, tobacco, and petunia. The phylogenetic tree was constructed with MEGA 7.0 program by the neighbor-joining method

An unrooted Neighbor-Joining tree was created for the characterization of the evolutionary relationships between *StTPSs* from potato and *TPSs* from tomato, pepper, tobacco and petunia (Fig. 6). Based on the phylogenetic tree (Fig. 6), these *TPS* genes could be classified into two main subfamilies (I and II), which is in agreement with previous work [44]. To determine the paralogous and orthologous relations among this family, the subfamily II *TPS* genes were further assigned to five groups (II-1, 2, 3, 4, and 5) with high bootstrap support. The number of potato, tomato, pepper, tobacco and petunia *TPS* genes in each of groups were I (0, 2, 2, 3, 2), II-1 (1, 1, 1, 2, 1) , II-2 (1, 1, 1, 2, 1) , II-3 (2, 1, 2, 2, 1) , II-4 (3, 3, 3, 5, 3) and II-5 (1, 1, 1, 3, 2) respectively. At least one gene of the five species was present in each group with the exception that no *StTPS* genes was in group I-1 (Fig. 6). The phylogenetic relationships among the five *Solanaceae* species suggested that genes in the same group may have similar function.

We were then interested to see if any amino acid substitutions in subgroups of TPSs have caused adaptive functional diversification. For this purpose, we evaluated the type I and type II functional divergence, between groups of the TPS family by posterior analysis [45] (Table 2). It was found that most type I coefficients (*θ*_I_) of functional divergence were significantly greater than zero (*P*<0.01), while few of the type II coefficients (*θ*_II_) were statistically greater than zero, implying that type I functional divergence was the dominant pattern for the evolution of TPS family in these plants. The results also showed that site-specific selective constraints on most members of TPS family may contribute to a group-specific functional evolution after their diversification as the coefficients of all functional divergence (*θ*) values between these groups were less than 1. The group II-1/II-3 had the least *θ*_*I*_ value (0.001), revealed that the lowest evolutionary rate or site specific selective relaxation was between these two groups. By contrast, the theta value in group pair II-1/II-5 was the greatest (0.908), implying the largest divergence between them.

**Table 2:** Functional divergence between groups of the *TPS* gene family in plant

Type I					Type II	
	*Θ*_I_±SE^a^	LRT	*P*	*Q*_*k*_≥0.95^b^	*Θ*_II_±SE	*P*
II-1/II-2	0.68±0.228	8.914	0.003	0	0.137±0.033	0
II-1/II-3	0.001±0.022	0	0.964	0	0.17±0.04	0
II-1/II-4	0.246±0.196	1.564	0.211	0	0.074±0.049	0.136
II-1/II-5	0.908±0.229	15.691	0	10	0.113±0.047	0.015
II-1/I-1	0.896±0.2	20.122	0	14	0.577±0.038	0
II-2/II-3	0.574±0.187	9.46	0.002	0	0.147±0.042	0
II-2/II-4	0.516±0.161	10.256	0.001	0	0.089±0.051	0.079
II-2/II-5	0.386±0.166	5.401	0.02	0	0.08±0.048	0.098
II-2/I-1	0.784±0.15	27.432	0	1	0.566±0.039	0
II-3/II-4	0.153±0.112	1.876	0.171	0	0.041±0.053	0.432
II-3/II-5	0.437±0.142	9.488	0.002	0	0.042±0.05	0.398
II-3/I-1	0.857±0.132	42.513	0	18	0.613±0.039	0
II-4/II-5	0.366±0.102	12.788	0	0	0.05±0.058	0.386
II-4/I-1	0.639±0.1	40.46	0	4	0.586±0.044	0
II-5/I-1	0.726±0.127	32.685	0	0	0.595±0.042	0

^a^The coefficient of functional divergence between the two subgroups and its standard error

^b^The number of critical amino acid residues with posterior probability (Q_k_) >0.95

**Fig. 7:**
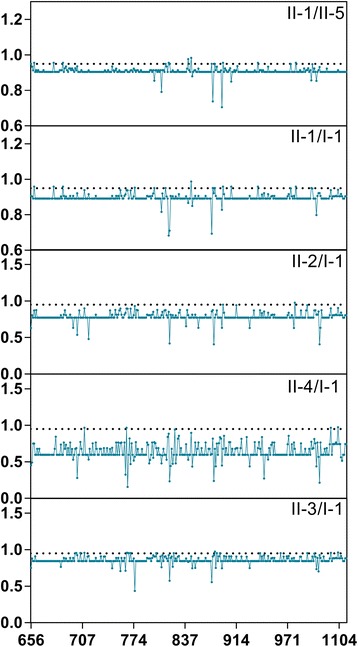
Type I functional divergence among the plant *TPS* gene family members. Posterior probability (PP) profiles of the site-specific type I functional divergence. The line indicates cutoff=0.95

To gain more information on the critical amino acid residues responsible for the functional divergence, all pairs of groups with functional divergence were used for posterior analysis. A cut off value (*Q*_*k*_≥0.95), as is frequently used in previous cited work [33], was used to identify type I functional divergence-related residues between groups. Most of the group pairs had at least one site in which the posterior probability was higher than 0.8. Among them, five pairs of groups had at least one site with posterior probability higher than 0.95 (Fig. 7). Similar to a previous report [33], the number and distribution of predicted sites for functional divergence within each pair are highly distinct. For example, only one critical amino acid site was predicted in the group II-2/I-1 pairs, while approximately 26 and 14 were predicted in the group II-3/I-1 and II-1/I-1 pairs, respectively. In total, 35 amino acid residues (656, 659, 679, 688, 700, 701, 705, 709, 767, 768, 769, 771, 775, 807, 809, 818, 821, 826, 840, 843, 865, 867, 869, 872, 877, 885, 935, 937, 964, 978, 979, 1069, 1073, 1096, 1103) in all comparisons were identified as being most important for the functional divergence (Fig. 7). It should be noted that all these amino acids were localized in the C-terminal region of TPSs.

Positive selection may be the most common factor that determines the retention of new genes after the duplication events, as many duplicated genes have been lost from the genome. Positive selection helps to accelerate the fixation of advantageous amino acids mutations which enable plants to adapt to its environment. By using the ML methods and codon substitution models, the selective pressure between the six groups of *TPS* genes were evaluated via the likelihood ratio tests [46, 47].

The ω of all *TPS* genes was estimated as 0.143 using one-ratio model (M0) (Table 3), which suggested that, on average, the *TPS* genes of five *Solanaceae* plants are under strong purifying selection. We then detected positive selection acting on particular group using a branch model in which each clade had its own ω (Table 3). Although the LRT statistic suggested that the ω of groups II-1 and II-3 were significantly different from other groups, the ω estimates for groups II-1 and II-3 still showed that they appear to have undergone purifying selection (Table 3).

**Table 3:** Parameter estimates and likelihood ratio tests for the branch model

Model	*p* ^a^	LnL^b^	Estimates of Parameters	2△l^c^	df	*p*	Positively selected sites
M0 (one ratio model)	1	-47935.395	ω=0.143	-	-	-	None
Branch-specific model (Model 2: two ratios)							
Estimate ω for I-1	2	-47935.222	ω_0_=0.143, ω_I1_= 0.083	Model 2 Vs M0: 0.346	1	0.556	-
Estimate ω for II-1	2	-47930.020	ω_0_= 0.146, ω_II1_= 0.083	Model 2 Vs M0: 10.750	1	0.001	-
Estimate ω for II-2	2	-47924.924	ω_0_= 0.147, ω_II2_= 0.065	Model 2 Vs M0: 20.943	1	0.091	-
Estimate ω for II-3	2	-47935.383	ω_0_= 0.143, ω_II3_= 0.147	Model 2 Vs M0: 0.024	1	0.000	-
Estimate ω for II-4	2	-47934.740	ω_0_= 0.143, ω_II4_= 0.199	Model 2 Vs M0: 1.310	1	0.252	-
Estimate ω for II-5	2	-47935.385	ω_0_= 0.143, ω_II5_= 0.148	Model 2 Vs M0: 0.020	1	0.888	-

^a^The number of free parameters for the ω ratios

^b^Likelihood of the model

^c^2(l_1_-l_0_)

As positive selection is unlikely to affect all sites over prolonged time, we thus estimated the evolutionary forces acting on individual codon site, using site-specific likelihood models of codon substitution [48, 49]. We use three pairs of models, forming three LRTs: M1 (neutral) and M2 (selection), M0 (one ratio) and M3 (discrete), and M7 (beta) and M8 (beta & ω) [48, 49]. The results in Table 4 showed that model M2 is not significantly better than M1, although it suggested that 17.8% sites were nearly neutral with ω=1. The model M3 with K=5 suggested that 1.1% sites were under positive selection, and M3 model was significantly better than the one-ratio model. Model M8, which with an additional ω ratio estimated from the data, is significantly better than M7, also suggested that 5.3% sites were under positive selection. Model M3 and M8 identified 1 and 3 amino acid sites under positive selection at the 95% cutoff.

**Table 4:** Parameter estimates and likelihood ratio tests for the site models

Model	*p* ^a^	LnL^b^	Estimates of Parameters	2△l^c^	df	*p*	Positively selected sites
M0 (one ratio model)	1	-47935.395	ω=0.143	-	-	-	None
Site-specific models							
M1: Neutral (k=2)	1	-47271.650	*p*_*0*_=0.774, (*p*_*1*_=0.226)	-	-	-	Not allowed
M2 : Selction (k=3)	3	-47271.650	*p*_*0*_=0.774, *p*_*1*_=0.048, (*p*_2_=0.178), ω_2_=1.000	M2 vs M1: 0.000	1	1.000	None
M3: discrete (K=5)	5	-46728.169	*p*_*0*_=0.244, *p*_*1*_=0.389, *p*_*2*_=0.263, *p*_*3*_=0.094, *p*_*4*_=0.011, ω_0_=0.010, ω_1_=0.085, ω_2_=0.317, ω_3_=0.799, ω_4_=6.007	M3 vs M0: 2414.452	4	0.000	1 (*p*>0.95)
M7: beta	2	-46761.572	*p*=0.513, *q*=1.910	-	-	-	Not allowed
M8: beta & w>1	4	-46732.126	*p*_*0*_=0.947, *p*=0.589, *q*=2.892, (*p*_*1*_=0.053) ω=1.293	M8 vs M7: 58.891	2	0.000	6 (*p*>0.95), 3 (*p*>0.95)

^a^The number of free parameters for the ω ratios

^b^Likelihood of the model

^c^2(l_1_-l_0_)

**Fig. 8:**
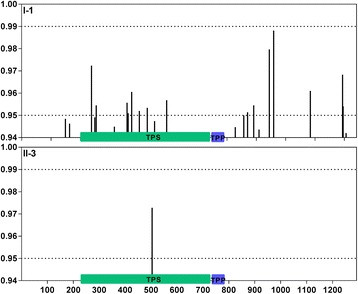
The Bayes Empirical Bayes (BEB) probabilities for sites in the positively selected class (ω>1). The x-axis denotes position in the amino acid alignment

To enhance the power in detecting positive selection, the branch-site model [50] was also applied to evaluate the selection on a few amino acids of TPS genes at specific groups (Table 5). LRT test showed that model A fit the data significantly better than the site-specific model M1 (*p*<0.01) in groups I-1, II-3, 4 and 5. Model A suggested positive selection on 46.1%, 4.6%, 8.5% and 7.2% sites of *TPS* genes in groups I-1, II-3, 4 and 5 respectively. At the posterior probabilities (p) >95% level, there were 15 and 1 sites identified which were likely to be under positive selection along the groups I-1 and II-3 respectively (Fig. 8). Referring to first sequence of StTPS3, these positively selected sites were 293H, 255L, 360Q, 364S, 376V, 402R, 429D, 495E, 771S, 792P, 845W, 860Y, 985Q, 1095D and 1097S in group I-1 and 504 F in group II-3. It is interesting to note that half of the positively selected sites in group I-1 and the only site identified in II-3 appeared in TPS domains, which suggests that these positively selected sites might cause adaptive changes after gene duplications that separated into different groups.

**Table 5:** Parameter estimates and likelihood ratio tests for the branch-site models

Model	*p* ^a^	LnL^b^	Estimates of Parameters	2△l^c^	df	*p*	Positively selected sites
M1: Neutral (k=2)	1	-47271.650	*p*_*0*_=0.774, (*p*_*1*_=0.226)				
Branch-site models							
Model A (I-1)	3	-47231.883	*p*_*0*_ *=* 0.416, *p*_*1*_ *=* 0.123, (*p*_*2a*_+*p*_*2b*_=0.461), ω_2_ *=1.291*	Model A vs M1: 79.535	2	0.000	Site for foreground lineage: 15 (at *p*>0.95)
Model A (II-1)	3	-47271.650	*p*_*0*_=0.774, *p*_*1*_= 0.226, (*p*_*2a*_+*p*_*2b*_ =0.000), ω_2_=2.639	Model A vs M1: 0.000	2	1.000	
Model A (II-2)	3	-47271.650	*p*_*0*_ *= 0.774*, *p*_*1*_ *= 0.226*, (*p*_*2a*_+*p*_*2b*_=0.000), ω_2_ *=1.000*	Model A vs M1: 0.000	2	1.000	
Model A (II-3)	3	-47253.663	*p*_*0*_=0.740, *p*_*1*_= 0.214, (*p*_*2a*_+*p*_*2b*_ =0.046), ω_2_=28.900	Model A vs M1: 35.974	2	0.000	Site for foreground lineage: 1 (at *p*>0.95)
Model A (II-4)	3	-47264.889	p_0_=0.707, p_1_= 0.207, (*p*_*2a*_+*p*_*2b*_ =0.085), ω_2_=1.506	Model A vs M1: 13.523	2	0.001	
Model A (II-5)	3	-47257.978	*p*_*0*_=0.718,*p*_*1*_= 0.210, (*p*_*2a*_+*p*_*2b*_ =0.072), ω_2_=3.022	Model A vs M1: 27.345	2	0.000	

^a^The number of free parameters for the ω ratios

^b^Likelihood of the model

^c^2(l_1_-l_0_)

### Expression pattern of *TPS* gene family in potato

*TPS* genes are known to be important in plants response to environmental stresses. In this study, we took advantage of available transcriptome data of potato, to analyze the complete set of *StTPS* genes in various tissues and under different phytohormones and abiotic stresses [51]. Transcripts of all *StTPS* gene family members were detected in all tested tissues of potato, although their abundance varied considerably.

**Fig. 9:**
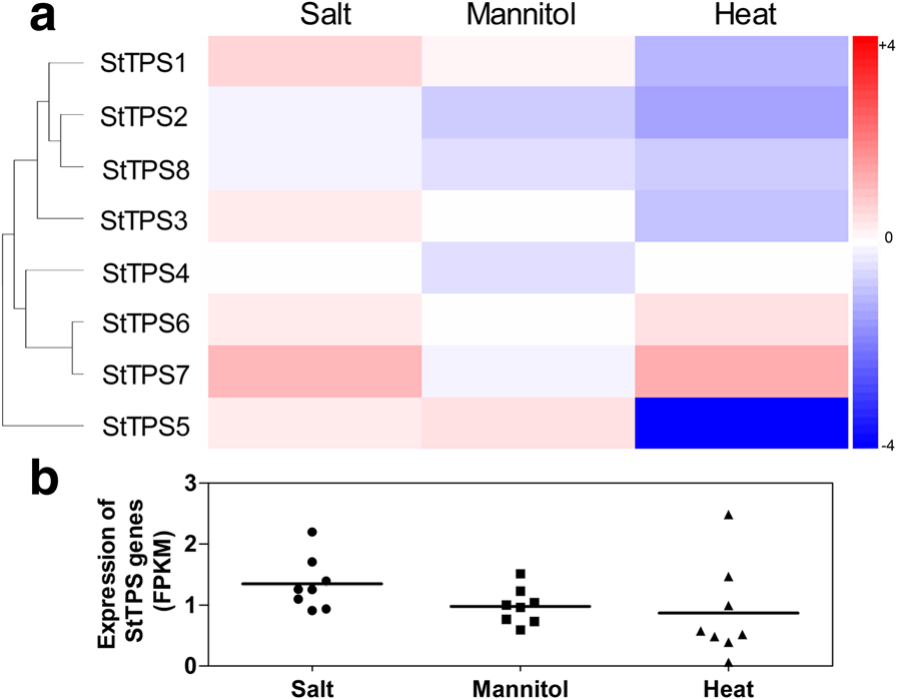
Expression profiles of the three *StTPS* genes upon different abiotic stresses. **a** Abiotic stress conditions (24-h treatment of in vitro grown whole plants) consisted of heat (35°C), salt (150 mM NaCl), and mannitol (260 mM) treatment. Control plants were grown in parallel with each stress treatment. Color scale represents fold changes (Treatment/Control) normalized log_2_ transformed data. Red indicates upregulated and blue indicates downregulated genes. **b** The scatter plot figure shows the FPKM value of each genes

Much work has been done in transgenic plants indicating that expressed *TPS* genes usually conferred higher tolerance to abiotic stresses [20, 52, 53]. In accordance with this, we found that *StTPS* genes showed differential expression patterns under various abiotic stresses (Fig. 9a). Under salt treatment, most *StTPS* genes were induced, whereas *StTPS2* and *StTPS8* were slightly downregulated. Under osmotic treatment (mannitol), only *StTPS1* and *StTPS5* exhibited increased expression. In contrast to salt stress, heat stress caused a large decline in transcriptional levels of most *StTPS* genes (*StTPS5* in particular), whereas *StTPS6* and *StTPS7* exhibited obvious increases in response to heat stress. *StTPS4* did not show obvious trends after heat treatment. Genes from the same group frequently showed similar expression pattern in various tissues. Based on the FPKM of different genes, the total transcript abundance of *StTPS* genes were highest in response to salt stress (Fig. 9b). Previous studies showed that *TPS* genes in maize were also upregulated in response to both salt and temperature stresses [43]. Enhanced *TPS* genes expression was observed for some “Resurrection plants”, in response to extreme water deficit, where up to 99% of their water has been removed. Thus, it is not surprising that *StTPS* genes were induced in potato upon water deficit caused by salt, mannitol and heat stresses.

**Fig. 10:**
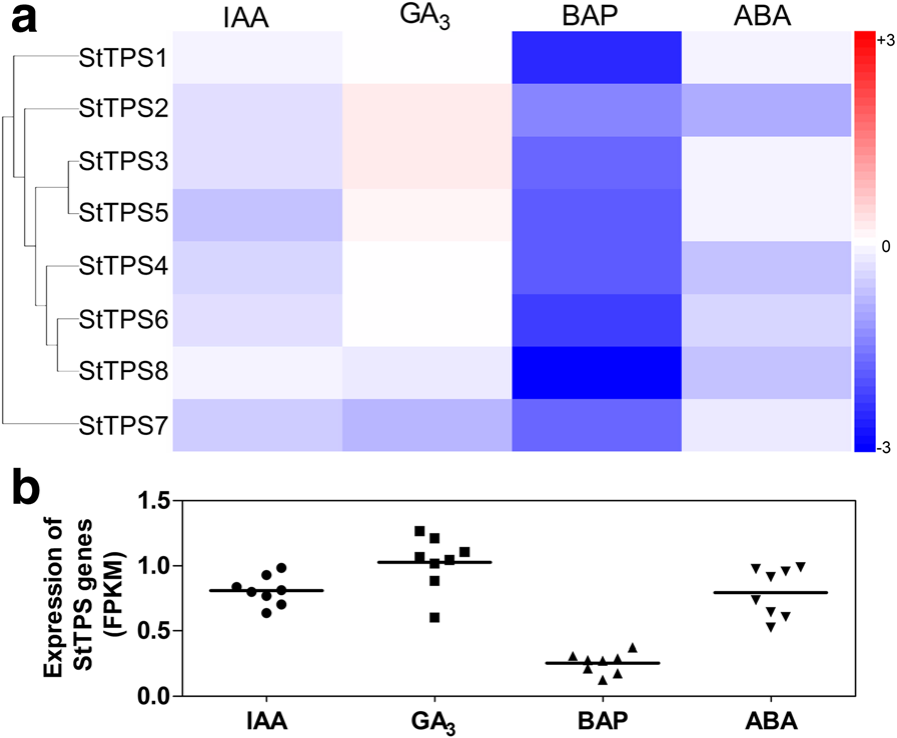
Expression profiles of the three *StTPS* genes upon different phytohormone treatment. **a** Hormone stress responses of in vitro grown whole plants were abscisic acid (ABA) (50 mM), indole-3-acetid acid (IAA) (10 mM), gibberellic acid (GA_3_) (50 mM), and 6-benzylaminopurine (BAP) (10 mM). Control plants were grown in parallel with each hormone treatment. Color scale represents fold changes (Treatment/Control) normalized log_2_ transformed data. Red indicates upregulated and blue indicates downregulated genes. **b** The scatter plot figure shows the FPKM value of each genes

Phytohormones play crucial roles in coordinating regulatory networks and the signal transduction pathways associated with external stimuli. In potato, we found that under various phytohormones treatments including abscisic acid (ABA), 6-benzylaminopurine (BAP), gibberellic acid (GA_3_), and indole-3-acetic acid (IAA), almost all the potato *TPS* genes were differentially downregulated except *StTPS2*, *StTPS3* and *StTPS5* which were slightly induced under GA treatment (Fig. 10a). The total transcript abundance of *StTPS* genes were extremely low in BAP treatment seedlings. BAP might be a key negative regulator of TPS abundance (Fig. 10b).

**Fig. 11:**
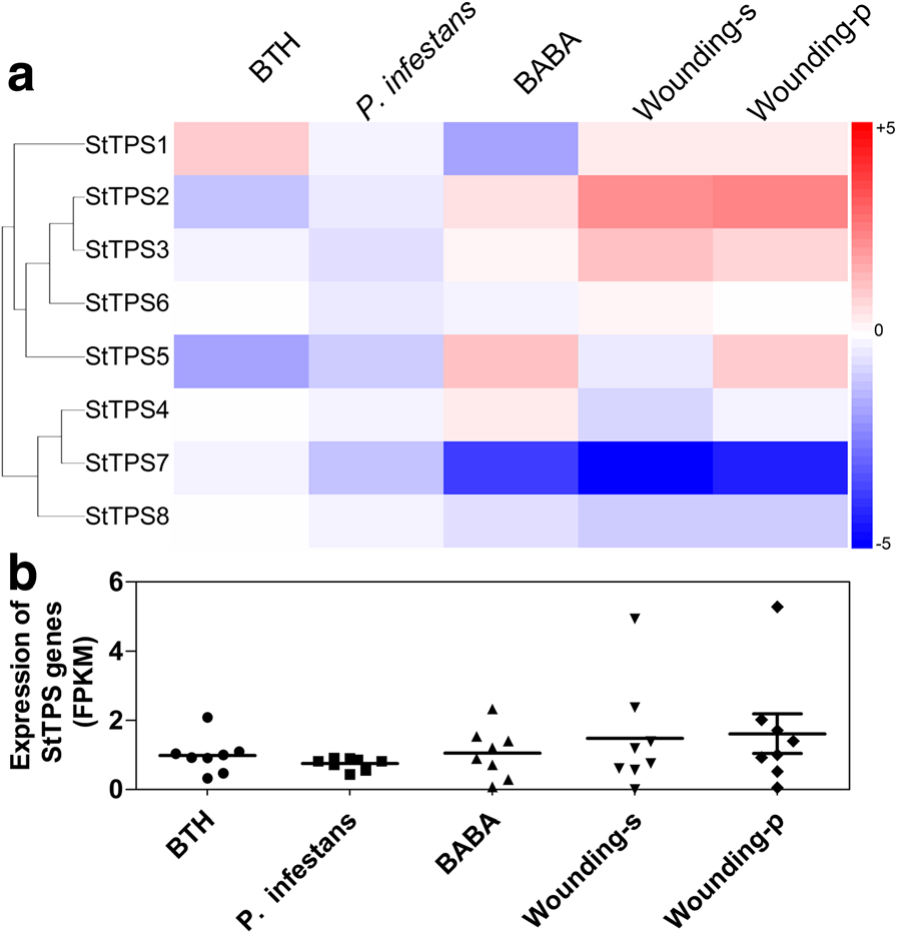
Expression profiles of the three *StTPS* genes upon different biotic stress or treatments. **a** The biotic stress conditions were induced with *Phytophthora infestans* inoculum, acibenzolar-S-methyl (BTH, 100 mg/ml) and DL-bamino-n-butyric acid (BABA, 2 mg/ml) using detached leaves. Mock inoculations were performed with sterile water. Color scale represents fold changes (Treatment/Control) normalized log_2_ transformed data. Red indicates upregulated and blue indicates downregulated genes. **b** The scatter plot figure shows the FPKM value of each genes

The expression data of *TPS* genes under various biotic stresses including leaves challenged with *Phytophthora infestans*, leaves wounded to mimic herbivory, and the elicitors acibenzolar-smethyl (BTH) and DL-ß-amino-n-butyric acid (BABA) were analyzed. *BABA* and BTH are well accepted inducers of resistance against pathogen infection. Under BTH and BABA treatment, five *TPS* genes including *StTPS1*, *StTPS2*, *StTPS3*, *StTPS4*, and *StTPS5* were differently induced. BTH and BABA exhibited differing effects on these five genes (Fig. 11a). For example, BTH could induce the expression of *StTPS1*, while BABA downregulated its expression level. As for the other four genes, BABA could induce expression of them when BTH downregulated. Upon *Phytoph*thora *infestans* infection, all the *StTPS* genes showed slightly decreased expression. Overall, either *Phytophthora infestans* infection or elicitors treatment showed less effect on *StTPS* genes. However, wounding leaves which mimicked herbivory caused obvious changes on expression of *StTPS* genes, especially on *StTPS2* and *StTPS7* genes. Wounding induced expression of *StTPS2* and *StTPS3*. Under all of these biotic stresses, *StTPS7* and *StTPS8* were always downregulated. However, the total transcript abundance of *StTPS* genes in wound treatment were obviously higher than other treatments (Fig. 11b).

**Fig. 12:**
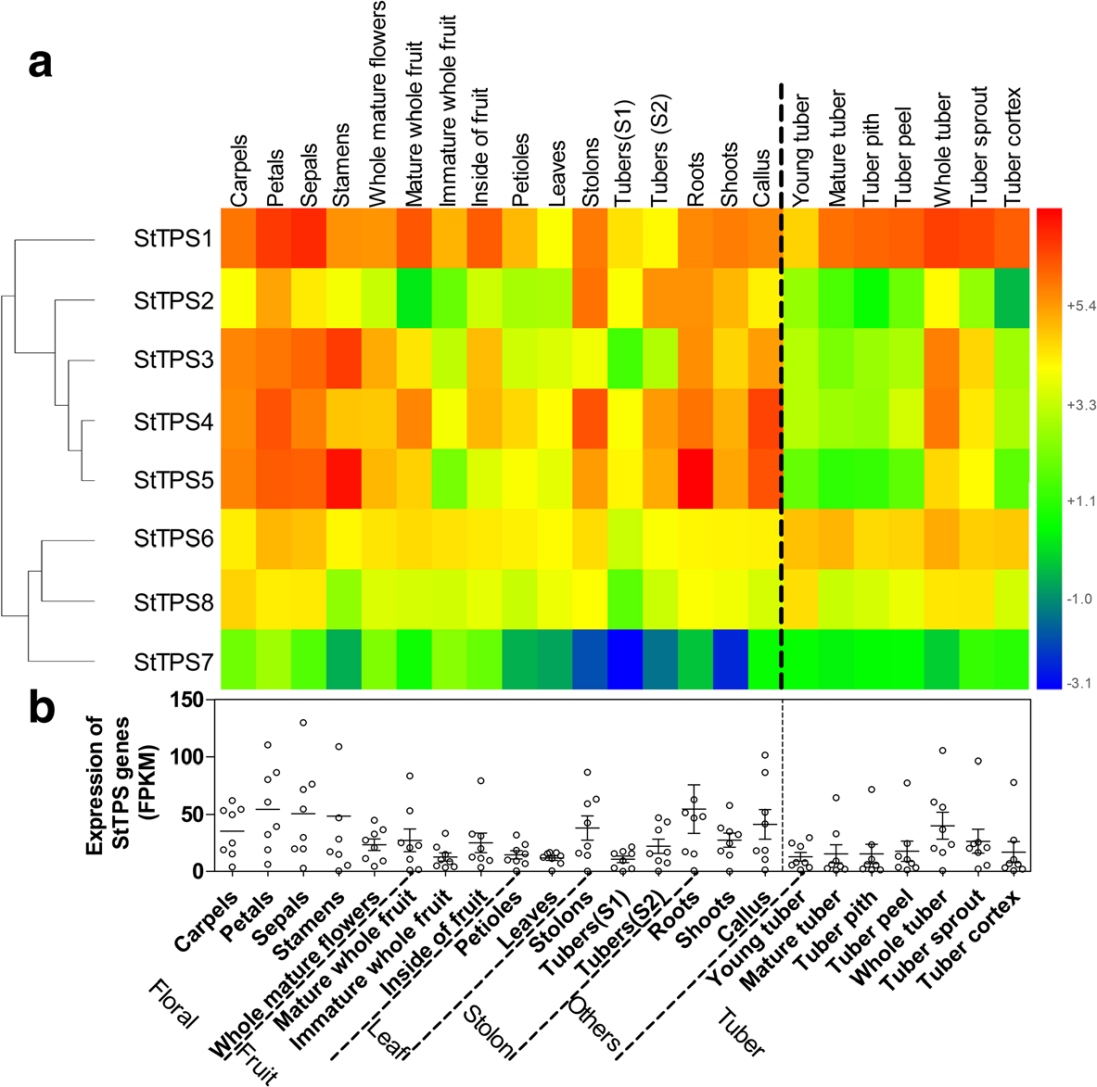
Expression profiles of the three *StTPS* genes upon different biotic stress or treatment. **a** The developmental tissues represent vegetative (leaves, petioles, stolons, tubers sampled twice) and reproductive organs (Floral: carpels, petals, sepals, stamens, whole flowers; Fruit: mesocarp/endocarp, whole immature berries, whole mature berries) from greenhouse-grown plants. Shoots and roots from in vitro-grown plants were also included in the developmental series. Callus (10–11 weeks old) derived from leaves and stems were used to assess transcription in an undifferentiated tissue. Color scale represents FPKM normalized log2 transformed data. Red indicates high expression level and blue indicates low expression level. **b** The scatter plot figure shows the FPKM value of each genes

Global gene expression analysis in various tissues revealed that *StTPS* genes were abundant in floral (stamens, sepals and petals) and root (average FPKM>40, four- fold higher than that in leaves) (Fig. 12). Moreover, *StTPS1* showed remarkably higher expression levels in almost every tissue, with average FPKM of 56 in different tissues, almost 28-fold higher than that of *StTPS7*, which has the lowest transcript level. Several studies using mutant plants have revealed the importance of trehalose metabolism in the control of plant development [7, 28, 54]. Moreover, there was some evidence showing that *AtTPS1* gene plays important roles in the control of stress response, cell and embryonic development, glucose sensing, and starch synthesis [7, 54, 55]. Beyond these established roles of *TPS* genes in plants, recent intriguing evidence has implicated these genes as important modulators of plant development and inflorescence architecture. Although less expressed, *StTPS7* was also found preferentially in floral tissues, indicating the role of *StTPS* genes in floral growth and development. Besides floral tissues, most *StTPS* genes show a slightly higher level of accumulation in root, shoot and callus. In different parts or growing stages of tuber, the general expression of *StTPS* genes are low except *StTPS1*, which showed relatively high expression levels in every part of the tuber.

### Validation of *StTPSs* differential expression

**Fig. 13:**
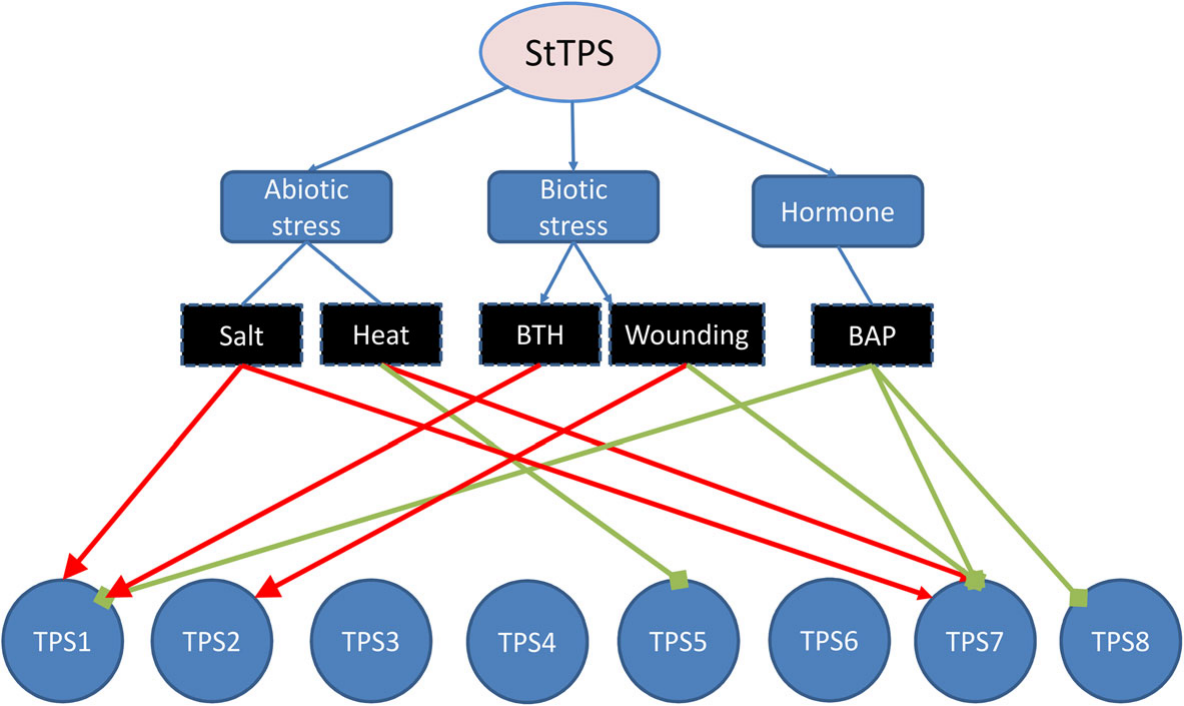
Validation of selected *StTPS* genes during exogenous stimuli. Abiotic stress conditions (24-h treatment of in vitro grown whole plants) consisted of heat (35°C), salt (150 mM NaCl) treatment. Control plants were grown in parallel with each stress treatment. The biotic stress condition (24 hr) was induced with acibenzolar-S-methyl (BTH, 100 mg/ml). Wounded leaves were included to mimic herbivory. Mock inoculations were performed with sterile water. The red line indicates the gene was upregulated, ca. two-fold greater than control. The green line represents the gene was down regulated, being less than 50% of control. The relative transcript abundance was normalized using potato actin gene

In *silico* analysis revealed that some *StTPS* genes are obviously regulated by different environmental stimuli. The differential expression of genes (fold changes >2) were chosen for qRT-PCR validation (Fig. 13). As expected, qRT-PCR results of genes under various treatment were similar in magnitude to those obtained by deep sequencing. qRT-PCR results suggested that two genes including *StTPS1* and *StTPS7* were frequently regulated by various treatments, which indicating they might be the primary TPSs involved in potato response to environment stimuli.

## Conclusions

In summary, we identified eight *StTPS* genes from potato and characterized their conserved protein motif, gene structure, chromosomal distribution, *cis*-acting elements in promoter regions and molecular evolution. Collectively, this has led to greater functional characterization of potato *TPS* genes. Moreover, analyses of their expression profiles based on available transcriptome data and qRT-PCR validation of various potato tissues under biotic and abiotic stress treatments provides functional information of *StTPSs*. Our results provide important clues for future research on the function of *StTPS* gene family and StTPS-mediated signal transduction pathways, thereby advancing our knowledge of the molecular basis of genetic enhancements to potato.

## Methods

### Identification and classification of *TPS* genes

To extensively identify potato *TPS* genes, Hidden Markov models (HMMs) of the ‘typical’ TPS and TPP domain were used to search the latest version of the potato genome (v4.04) from Spud DB [51] and the genomes of four other *Solanaceae* species including tomato (*Solanum lycopersicum*, v3.1, id35173 ), pepper (*Capsicum annuum*, v2, id22828), tobacco (*Nicotiana tabacum*, TN90), petunia (*Petunia_axillaris*, v0.1, id24480) via HMMER v. 3.1 [32] with an E-value cut-off of <1e-10. When several variants of one gene were obtained, only the longest one was retained. All the candidate sequences were further confirmed to have complete PFAM TPS and TPP domains. Pseudogenes which only covered less than 50% of the PFAM domain models were eliminated from final *TPS* genes [56]. The basic physical and chemical properties of the protein sequences were analyzed using ProParam online tool in ExPASy. Subcellular localization predictor (http://cello.life.nctu.edu.tw/) was used to predict subcellular localizations. The chromosomal locations of *TPS* genes were drawn based on the potato genome database deposited [51]. MCScanX was used to analyze the syntenic relationships within genomes of melon, watermelon and cucumber respectively [57]. The diagrams were visualized using Circos software (v0.67).

### Gene structure and conserved motifs analyses

Gene structures of *TPSs* were analyzed on the Gene Structure Display Server 2.0 (GSDS; http://gsds.cbi.pku.edu.cn/). Motifs in the candidate potato TPS protein sequences [58] were predicted using program MEME (http://meme-suite.org) with the default parameters.

### Sequence alignment and phylogenetic analysis

Sequence alignment among *TPS* genes from potato or different species were performed using the ClustalX 1.83 software with full-length CDS sequences of *TPS* genes [59] and the alignment results were displayed with DNAMAN v5.2. MEGA v7 was used to construct the phylogenetic tree using the Neighbor-Joining method [60]. The percentage of replicate trees in which the associated taxa clustered together in the bootstrap test (1000 replicates) [61].

### Functional divergence analyses

To estimate the level of functional divergence in the TPS subgroups, coefficients of Type-I and Type-II functional divergence were calculated using DIVERGE (version 2.0)X Gu [62].

### Selection assessment and testing

The values of nonsynonymous substitutions (*d*_*N*_), synonymous substitutions (*d*_*S*_) and *d*_*N*_/*d*_*S*_ ratio (or ω) were calculated via the program PAML version 4 [63], using branch-specific (model B), site-specific (neutral, selection, discrete, beta, beta & w>1), and branch-site models as implemented in PAML [50, 64]. Likelihood ratio test (LRT) were used to compare the fit of model pairs. The sites under positive selection were identified using Bayes methods [65].

### In silico expression analysis of *StTPSs*

Transcriptome gene expression data were extracted from NCBI sequence Read Archive (SRA029323) and Spud DB [51, 66–68] to analyze the expression profiles of potato in organs and under different treatments as described in figure legends. The reads were mapped to *S. tuberosum* Group Phureja DM1-3 super scaffolds using Tophat (v1.4.1). The FPKM values were calculated by Cufflinks (v1.3.0) using v3.4 representative model.

### Plant growth and treatments

Potato plants (*S. tuberosum* L. cultivar Shepody) were cultivated in a growth chamber in soil at 25 °C under a photoperiod of 16 h light/8 h dark. After growing for 30 d, the seedlings were used for treatments. Abiotic stress conditions (24-h treatment of in vitro grown whole plants) consisted of heat (35°C), salt (150 mM NaCl) treatment. Control plants were grown in parallel with each stress treatment. The biotic stress condition (24 h) was induced with acibenzolar-S-methyl (BTH, 100 mg/ml). Wounded leaves were included to mimic herbivory. Mock inoculations were performed with sterile water. After various treatments, the seedlings were sampled, then immediately frozen in liquid nitrogen, and stored at -80 °C until further analysis.

### Real-time quantitative PCR

Total RNA was extracted from each sample, which were first homogenized with mortar and pestle in liquid nitrogen, using TRIzol reagent (Invitrogen, USA) according to the instructions supplied by the manufacturer. About 4 μg of total RNA was reverse-transcribed using an oligo(dT) primer and SuperScript Reverse Transcriptase (Invitrogen, USA). Real-time quantitative PCR was conducted using SYBR green (TaKaRa Biotechnology) on Mastercycler® ep *realplex* real-time PCR system (Eppendorf, Hamburg, Germany). Gene-specific primers for each *StTPS* gene were designed using Primer Premier 5.0 and optimized using oligo 7. The relative abundance of *Actin 1* was used as the internal standard. Primer pairs included: *StTPS1* (F-5'TGATGTAGTTGCCGATGC3' R-5' GATTGCCCTGGTTGTTGT3'); *StTPS2* (F- 5' AAATACCGTGTTTCTCGT 3' R- 5' CCTTTACTGACTCCCTGA 3'); *StTPS5* (F- 5' AGGAAGGGATACGCTCAG 3' R- 5' CCAAATGCCAAAGTCAGG 3'); *StTPS7* (F- 5' GAACGGAGAAGCTGGATG 3' R- 5' CTCTGCCTCGGAGACAAT 3'); *StTPS8* (F- 5' CTCAAGGCTTTGCTCTGT 3' R- 5' GATGCCTACTGTCCTACCAT 3'); *Actin* (F- 5' CACCCTGTTCTGCTCACT 3' R- 5' CAGCCTGAATAGCAACATAC 3'). Real-time PCR reactions were performed in a total reaction volume of 25 μL using the following conditions: 94 °C /2 min; 40 cycles (94 °C /15 s, 58 °C /15 s, 72 °C /15 s). All reactions were performed in triplicate. Independent experiments were repeated three times. Relative gene expression was analyzed using the 2 ^-ΔΔc(t)^ method [65].

## Additional files

Additional file 1: Figure S1. Amino acid sequence alignment of potato TPS proteins. Strictly conserved sequence is in white on black background; similar amino acids are in black on green background. Residues involved in the catalytic center are placed in boxes. (DOCX 857 kb)

Additional file 2: Figure S2. Conserved motifs in StTPS proteins. (DOCX 440 kb)

## Abbreviations

ABA: Abscisic acid
ABREs: ABA-responsive elements
BABA: DL-ß-amino-n-butyric acid
BAP: 6-benzylaminopurine
BTH: Acibenzolar-smethyl
d_N_: Non-synonymous substitution rate
d_S_: Synonymous substitution rate
GA_3_: Gibberellic acid
IAA: Indole-3-acetic acid
T-6-P: Trehalose-6-P
TPP: T-6-P phosphatase
TPS: Trehalose-6-phosphate synthase

## References

[1] Chary SN, Hicks GR, Choi YG, Carter D, Raikhel NV (2008). Trehalose-6-phosphate synthase/phosphatase regulates cell shape and plant architecture in Arabidopsis. Plant Physiol..

[2] Lunn JE, Delorge I, Figueroa CM, Dijck PV, Stitt M (2014). Trehalose metabolism in plants. Plant J..

[3] Elbein AD, Pan YT, Pastuszak I, Carroll D (2003). New insights on trehalose: a multifunctional molecule. Glycobiology..

[4] Cai ZJ, Peng GX, Cao YQ, Liu YC, Jin K, Xia YX (2009). Trehalose-6-phosphate synthase 1 from Metarhizium anisopliae: clone, expression and properties of the recombinant. J Biosci Bioeng..

[5] López MF, Männer P, Willmann A, Hampp R, Nehls U (2007). Increased trehalose biosynthesis in Hartig net hyphae of ectomycorrhizas. New Phytol..

[6] Gibson RP, Tarling CA, Roberts S, Withers SG, Davies GJ (2004). The donor subsite of trehalose-6-phosphate synthase - Binary complexes with UDP-glucose and UDP-2-deoxy-2-fluoro-glucose at 2 angstrom resolution. J Biol Chem..

[7] van Dijken AJ, Schluepmann H, Smeekens SC (2004). Arabidopsis trehalose-6-phosphate synthase 1 is essential for normal vegetative growth and transition to flowering. Plant Physiol..

[8] Cabib E, Leloir LF (1958). The biosynthesis of trehalose phosphate. J Biol Chem..

[9] Márquez-Escalante JA, Figueroa-Soto CG, Valenzuela-Soto EM (2006). Isolation and partial characterization of trehalose 6-phosphate synthase aggregates from Selaginella lepidophylla plants. Biochimie..

[10] Valenzuela-Soto EM, Márquez-Escalante JA, Iturriaga G, Figueroa-Soto CG (2004). Trehalose 6-phosphate synthase from Selaginella lepidophylla : purification and properties. Biochem Biophys Res Commun..

[11] Londesborough J, Vuorio OE (1993). Purification of trehalose synthase from baker's yeast. Eur J Biochem..

[12] Pan YT, Carroll JD, Elbein AD (2002). Trehalose-phosphate synthase of Mycobacterium tuberculosis. Cloning, expression and properties of the recombinant enzyme. Eur J Biochem..

[13] Pan YT, Koroth EV, Jourdian WJ, Edmondson R, Carroll JD, Pastuszak I, Elbein AD (2004). Trehalose synthase of Mycobacterium smegmatis: purification, cloning, expression, and properties of the enzyme. Eur J Biochem..

[14] Deng YY, Wang XL, Guo H, Duan DLA (2014). trehalose-6-phosphate synthase gene from *Saccharina japonica* (Laminariales, Phaeophyceae). Mol Biol Rep..

[15] Thevelein JM (1992). The RAS-adenylate cyclase pathway and cell cycle control in Saccharomyces cerevisiae. Antonie Van Leeuwenhoek..

[16] Stiller I, Dulai S, Kodrak M, Tarnai R, Szabo L, Toldi O, Banfalvi Z (2008). Effects of drought on water content and photosynthetic parameters in potato plants expressing the trehalose-6-phosphate synthase gene of Saccharomyces cerevisiae. Planta..

[17] Romero C, Bellés JM, Vayá JL, Serrano R, Culiáñez-Macià FA (1997). Expression of the yeast trehalose-6-phosphate synthase gene in transgenic tobacco plants: pleiotropic phenotypes include drought tolerance. Planta..

[18] Mu M, XK L, Wang JJ, Wang DL, Yin ZJ, Wang S, Fan WL, Ye WW. Genome-wide Identification and analysis of the stress-resistance function of the TPS (Trehalose-6-Phosphate Synthase) gene family in cotton. BMC Genet. 2016;17 10.1186/s12863-016-0360-y PMC4797179 26993467

[19] Zentella R, Iturriaga GA (1999). *Selaginella lepidophylla* trehalose-6-phosphate synthase complements growth and stress-tolerance defects in a yeast tps1 mutant. Plant Physiol..

[20] Garg AK, Kim JK, Owens TG, Ranwala AP, Yang DC, Kochian LV, Trehalose WRJ (2002). accumulation in rice plants confers high tolerance levels to different abiotic stresses. Proc Natl Acad Sci U S A..

[21] In-Cheol Jang S-JO, Seo J-S, Choi W-B, Song SI, Kim CH, Kim YS, Seo H-S, Do Choi Y, Nahm BH, Kim J-K (2003). Expression of a bifunctional fusion of the Escherichia coli genes for trehalose-6-phosphate synthase and trehalose-6-phosphate phosphatase in transgenic rice plants increases trehalose accumulation and abiotic stress tolerance without stunting growth. Plant Physiol..

[22] Zang B, Li H, Li W, Deng XW, Wang X (2011). Analysis of trehalose-6-phosphatesynthase (TPS) gene family suggests the formation of TPS complexes in rice. Plant Mol Biol. Plant Mol Bio..

[23] Avonce N, Mendoza-Vargas A, Morett E, Iturriaga G (2006). Insights on the evolution of trehalose biosynthesis. BMC Evol Biol.

[24] Lunn JE (2007). Gene families and evolution of trehalose metabolism in plants. Functional Plant Biology..

[25] Leyman B, Dijck PV, Thevelein JM (2001). An unexpected plethora of trehalose biosynthesis genes in Arabidopsis thaliana. Trends Plant Sci..

[26] Vandesteene M, Ramon K, Patrick D, Filip R (2010). A single active trehalose-6-p synthase (TPS) and a family of putative regulatory TPS-Like proteins in Arabidopsis. Molecular Plant..

[27] RAMON M, ID SMET, Vandesteene L, Naudts M, LEYMAN B, Dijck PV, ROLLAND F, Beeckman T, Thevelein JM (2009). Extensive expression regulation and lack of heterologous enzymatic activity of the Class II trehalose metabolism proteins from Arabidopsis thaliana. Plant Cell Environ..

[28] Avonce N, Leyman B, Mascorrogallardo JO, Dijck PV, Thevelein JM, Iturriaga G (2004). The *Arabidopsis* trehalose-6-P synthase AtTPS1 gene is a regulator of glucose, abscisic acid, and stress signaling. Plant Physiol..

[29] Wang GL, Zhao G, Feng YB, Xuan JS, Sun JW, Guo BT, Jiang GY, Weng ML, Yao JT, Wang B (2010). Cloning and comparative studies of seaweed trehalose-6-phosphate synthase genes. Mar Drugs..

[30] Zhang Y, Primavesi LF, Jhurreea D, Andralojc PJ, Mitchell RA, Powers SJ, Schluepmann H, Delatte T, Wingler A, Paul MJ (2011). Inhibition of SNF1-related protein kinase1 activity and regulation of metabolic pathways by trehalose-6-phosphate. Chin Sci Bull..

[31] Cai Z, Peng G, Cao Y, Liu Y, Kai J, Xia Y (2009). Trehalose-6-phosphate synthase 1 from Metarhizium anisopliae: clone, expression and properties of the recombinant. J Biosci Bioeng..

[32] Eddy SRA. new generation of homology search tools based on probabilistic inference. Genome Informatics International Conference on. Genome Informatics. 2009:205–11. 20180275

[33] Yang HL, Liu YJ, Wang CL, Zeng QY (2012). Molecular evolution of trehalose-6-phosphate synthase (TPS) gene family in Populus, Arabidopsis and rice. PLoS One..

[34] Consortium PGS, Xu X, Pan S, Cheng S, Zhang B, Mu D, Ni P, Zhang G, Yang S, Li R (2011). Genome sequence and analysis of the tuber crop potato. Nature..

[35] Song J, Gao ZH, Huo XM, Sun HL, YS X, Shi T, Ni ZJ. Genome-wide identification of the auxin response factor (ARF) gene family and expression analysis of its role associated with pistil development in Japanese apricot (Prunus mume Sieb. et Zucc). Acta Physiologiae Plantarum. 2015;37(8)

[36] Wang X, Shi X, Hao B, Ge S, Luo J (2005). Duplication and DNA segmental loss in the rice genome: implications for diploidization. New Phytol..

[37] Zhang Y, Mao L, Wang H, Brocker C, Yin X, Vasiliou V, Fei Z, Wang X (2012). Genome-wide identification and analysis of grape aldehyde dehydrogenase (ALDH) gene superfamily. PLoS One..

[38] Mu M, X-K L, Wang J-J, Wang D-L, Yin Z-J, Wang S, Fan W-L, Ye W-W (2016). Genome-wide Identification and analysis of the stress-resistance function of the TPS (Trehalose-6-Phosphate Synthase) gene family in cotton. BMC Genet..

[39] Doi K, Hosaka A, Nagata T, Satoh K, Suzuki K, Mauleon R, Mendoza MJ, Bruskiewich R, Kikuchi S. The development of a novel data mining tool to find ciselements in rice gene promoter regions. BMC Plant Biol. 2008;8:20. 10.1186/1471-2229-8-20 PMC2270273 18302796

[40] Cao JM, Jiang M, Li P, Chu ZQ. Genome-wide identification and evolutionary analyses of the PP2C gene family with their expression profiling in response to multiple stresses in Brachypodium distachyon. BMC Genomics. 2016;17:175. 10.1186/s12864-016-2526-4 PMC4776448 26935448

[41] Li W, Liang W (2012). Transcriptional regulation of Arabidopsis MIR168a and argonaute1 homeostasis in abscisic acid and abiotic stress responses. Plant Physiol..

[42] Kosmas SA, Argyrokastritis A, Loukas MG, Eliopoulos E, Tsakas S, Kaltsikes PJ (2006). Isolation and characterization of drought-related trehalose 6-phosphate-synthase gene from cultivated cotton (*Gossypium hirsutum* L.). Planta..

[43] Jiang W, FL F, Zhang SZ, Wu L, Li WC (2010). Cloning and Characterization of Functional Trehalose-6-Phosphate Synthase Gene in Maize. Journal of Plant Biology..

[44] Zang B, Li H, Li W, Deng XW, Wang X (2011). Analysis of trehalose-6-phosphatesynthase (TPS) gene family suggests the formation of TPS complexes in rice. Plant Mol Biol. Plant Mol Biol..

[45] Gu X, Velden KVDIVERGE (2002). phylogeny-based analysis for functional-structural divergence of a protein family. Bioinformatics..

[46] Yang Z, Bielawski JP (2000). Statistical methods for detecting molecular adaptation. Trends Ecol Evol..

[47] Yang HL, Liu YJ, Wang CL, Zeng QY (2012). Molecular evolution of trehalose-6-phosphate synthase (TPS) gene family in Populus, Arabidopsis and Rice. PLoS One..

[48] Nielsen R, Yang Z (1998). Likelihood models for detecting positively selected amino acid sites and applications to the HIV-1 envelope gene. Genetics..

[49] Yang Z, Nielsen R, Goldman N, Pedersen AM (2000). Codon-substitution models for heterogeneous selection pressure at amino acid sites. Genetics..

[50] Yang Z, Nielsen R (2002). Codon-Substitution Models for Detecting Molecular Adaptation at Individual Sites Along Specific Lineages. Mol Biol Evol..

[51] Hardigan MA, Crisovan E, Hamiltion JP, Kim J, Laimbeer P, Leisner CP, Manrique-Carpintero NC, Newton L, Pham GM, Vaillancourt B (2016). Genome reduction uncovers a large dispensable genome and adaptive role for copy number variation in asexually propagated Solanum tuberosum. Plant Cell..

[52] Ge LF, Chao DY, Shi M, Zhu MZ, Gao JP, Lin HX (2008). Overexpression of the trehalose-6-phosphate phosphatase gene OsTPP1 confers stress tolerance in rice and results in the activation of stress responsive genes. Planta..

[53] Miranda JA, Avonce N, Suárez R, Thevelein JM, Dijck PV, Iturriaga GA (2007). bifunctional TPS-TPP enzyme from yeast confers tolerance to multiple and extreme abiotic-stress conditions in transgenic Arabidopsis. Planta..

[54] Gilday A, Li Y, Graham IA (2006). Delayed embryo development in the ARABIDOPSIS TREHALOSE-6-PHOSPHATE SYNTHASE 1 mutant is associated with altered cell wall structure, decreased cell division and starch accumulation. Plant J..

[55] Gilday A, Feil R, Lunn JE, Graham IA. AtTPS1-mediated trehalose 6-phosphate synthesis is essential for embryogenic and vegetative growth and responsiveness to ABA in germinating seeds and stomatal guard cells. Plant J. 2010;64:1–13. 10.1111/j.1365-313X.2010.04312.x 20659274

[56] Lehti-Shiu MD, Shiu SH. Diversity, classification and function of the plant protein kinase superfamily. Philos Trans R Soc Lond. 2012;367(1602):2619–39. 10.1098/rstb.2012.0003 PMC3415837 22889912

[57] Wang Y, Tang H, Debarry JD, Tan X, Li J, Wang X, Lee TH, Jin H, Marler B, Guo H (2012). MCScanX: a toolkit for detection and evolutionary analysis of gene synteny and collinearity. Nucleic Acids Res..

[58] Bailey TL, Williams N, Misleh C, Li WWMEME (2006). discovering and analyzing DNA and protein sequence motifs. Nucleic Acids Res..

[59] Chenna R, Sugawara H, Koike T, Lopez R, Gibsom TJ, Higgins DG, Thompson JD (2003). Multiple sequence alignment with the Clustal series of programs. Nucleic Acids Res..

[60] Saitou N, Nei M (1987). The neighbor-joining method: a new method for reconstructing phylogenetic trees. Molbiolevol..

[61] Jones DT, Taylor WR, Thornton JM (1992). The rapid generation of mutation data matrices from protein sequences. Computer Applications in the Biosciences Cabios..

[62] Maximum-likelihood GX (2001). approach for gene family evolution under functionaldivergence. Mol Biol Evol..

[63] Zhao Y, Fu L, Li R, Wang LN, Yang Y, Liu NN, Zhang CM, Wang Y, Liu P, PAML TBB (2007). 4: phylogenetic analysis by maximum likelihood. Mol Biol Evol..

[64] Yang Z, Nielsen R (1998). Synonymous and nonsynonymous rate variation in nuclear genes of mammals. J Mol Evol..

[65] Yang Z, Wong WS, Nielsen R (2005). Bayes empirical bayes inference of amino acid sites under positive selection. Mol Biol Evol..

[66] Xu X, Pan S, Cheng S, Zhang B, Mu D, Ni P, Zhang G, Yang S, Li R, Wang J (2011). Genome Sequence and Analysis of the Tuber Crop Potato. Nature..

[67] Massa AN, Childs KL, Buell CR (2013). Abiotic and Biotic Stress Responses in Solanum tuberosum Group Phureja DM1-3 516 R44 as Measured through Whole Transcriptome Sequencing. Plant Genome..

[68] Massa AN, Childs KL, Lin H, Bryan GJ, Giuliano G, Buell CR. The transcriptome of the reference potato genome *Solanum tuberosum* Group Phureja Clone DM1-3 516R44. PLoS ONE. 2011;6(10):e26801. 10.1371/journal.pone.0026801 PMC3203163 22046362

