# Supplementary material for: Genome-wide analysis of the Solanum tuberosum (potato) trehalose-6-phosphate synthase (TPS) gene family: evolution and differential expression during development and stress
Source: BMC Genomics. 2017 Dec 1;18:926. doi: 10.1186/s12864-017-4298-x (PMC5710090; doi:10.1186/s12864-017-4298-x)
Supplement: Supplementary file 2 — Conserved motifs in StTPS proteins. (DOCX 440 kb) [file 12864_2017_4298_MOESM2_ESM.docx]

**Genome-wide analysis of the *Solanum tuberosum* (potato) trehalose-6-phosphate synthase (TPS) gene family: evolution and differential expression during development and stress**

**Yingchun Xu^a^, Liu Yang^b^, Neil Mattson^c^, Yanjie Wang^a^, Qijiang Jin^a*^**

a College of Horticulture, Nanjing Agricultural University, Nanjing 210095, P.R. China

b Institute of Plant Protection, Jiangsu Academy of Agricultural Sciences, Nanjing 210095, China

**c Horticulture Section, School of Integrative Plant Science, Cornell University, 134A Plant Science Bldg, Ithaca, NY 14853, USA**

.

***Corresponding author**

**E-mail:** jqj@njau.edu.cn;

Mail address: College of Horticulture, Nanjing Agricultural University, Nanjing 210095, China


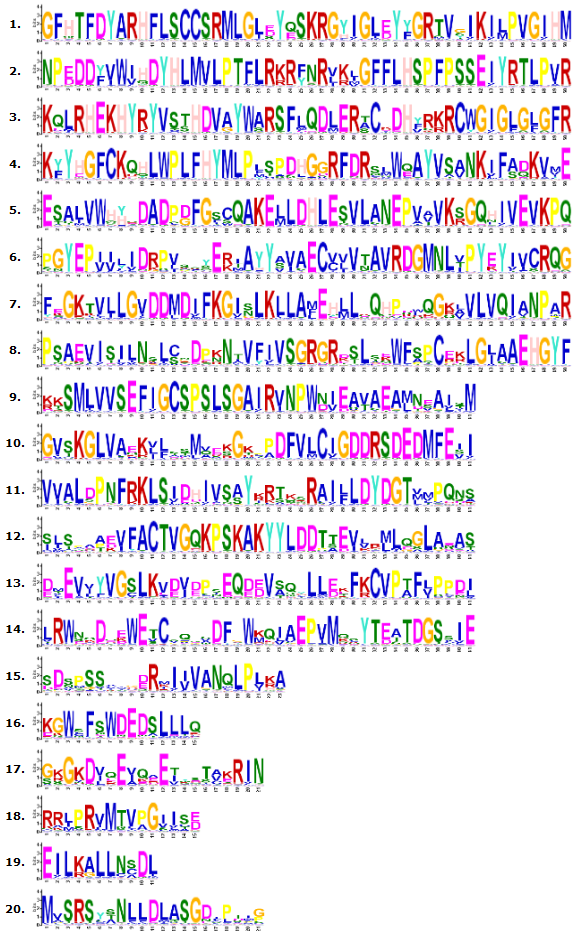
 Supplementary Figure S2 Detail information of identified motifs.
